# Serum interleukin-6, tumour necrosis factor-a, D-dimer after recombinant human brain natriuretic peptide combined with levosimendan in patients with heart failure

**DOI:** 10.5937/jomb0-56202

**Published:** 2025-08-21

**Authors:** Yujing Zhang, Pingyan Fei, Xinyu Liu

**Affiliations:** 1 Songjiang Hospital Affiliated to Shanghai Jiao Tong University School of Medicine, Department of Cardiology, Shanghai, China

**Keywords:** heart failure, recombinant human brain natriuretic peptide (Rh-BNP), levosimendan, inflammatory markers, nutritional support, cardiac function, quality of life, srcana insuficijencija, rekombinovani ljudski moždani natriuretski peptid (Rh-BNP), levozimendan, inflamatorni markeri, nutritivna podrška, srcana funkcija, kvalitet života

## Abstract

**Background:**

This study evaluates the impact of recombinant human brain natriuretic peptide (Rh-BNP) combined with levosimendan on therapeutic efficacy, inflammatory markers, and nutritional status in patients with heart failure (HF).

**Methods:**

A total of 162 heart failure patients treated between March 2022 and March 2024 were randomly assigned to three treatment groups: Group 1 received conventional therapy plus Rh-BNP Group 2 received conventional therapy plus levosimendan, and Group 3 received a combination of both Rh-BNP and levosimendan, along with nutritional support. Efficacy was assessed regarding cardiac function, renal function, inflammatory biomarkers (IL-6, TNF-a), nutritional status, and quality of life.

**Results:**

All three treatment groups improved cardiac and renal function, reduced inflammatory markers, and enhanced nutritional status. However, the combination therapy group (Group 3) demonstrated the most significant improvements across all parameters, including a greater reduction in IL-6 and TNF-a levels and an improvement in the Mini Nutritional Assessment (MNA) score. Additionally, the combination treatment led to marked improvements in quality of life and reduced symptoms of depression and anxiety compared to the other groups (P< 0.05).

**Conclusions:**

Rh-BNP combined with levosimendan, alongside nutritional support, offers significant benefits in treating heart failure, enhancing cardiac function, reducing inflammation, improving nutritional status, and improving the overall quality of life for patients.

## Abstract (sr)

**Uvod:**

Ova studija procenjuje uticaj rekombinovanog humanog moždanog natriuretskog peptida (Rh-BNP) u kombinaciji sa levozimendanom na terapijsku efikasnost, inflamatorne markere i nutritivni status kod pacijenata sa srčanom insuficijencijom (HF).

**Metode:**

Ukupno 162 pacijenta sa srčanom insuficijen-cijom, lečena u periodu od marta 2022. do marta 2024. godine, su nasumično raspoređena u tri terapijske grupe: Grupa 1 je primala konvencionalnu terapiju uz Rh-BNP Grupa 2 konvencionalnu terapiju uz levozimendan, dok je Grupa 3 dobijala kombinaciju Rh-BNP i levozimendana, uz nutritivnu podršku. Efikasnost terapije procenjivana je na osnovu srčane i bubrežne funkcije, inflamatornih biomar-kera (IL-6, TNF-a), nutritivnog statusa i kvaliteta života.

**Rezultati:**

Sve tri terapijske grupe pokazale su poboljšanje srčane i bubrežne funkcije, smanjenje inflamatornih mar-kera i poboljšanje nutritivnog statusa. Međutim, grupa sa kombinovanom terapijom (Grupa 3) pokazala je najzna-čajnija poboljšanja u svim parametrima, uključujući veće smanjenje nivoa IL-6 i TNF-a i poboljšanje rezultata Mini nutritivne procene (MNA). Pored toga, kombinovana te-rapija dovela je do značajnog poboljšanja kvaliteta života i smanjenja simptoma depresije i anksioznosti u poređenju sa ostalim grupama (P< 0,05).

**Zaključak:**

Rh-BNP u kombinaciji sa levozimendanom, uz nutritivnu podršku, pruža znacajne prednosti u lecenju srcane insuficijencije, poboljšavajući srcanu funkciju, smanjujući inflamaciju, unapređujući nutritivni status i poboljšavajući celokupni kvalitet života pacijenata

## Introduction

Heart failure (HF) is a prevalent and complex clinical syndrome that affects millions of individuals globally [1]. As of 2017, there were an estimated 64.3 million cases worldwide [2] [3] [4] [5], and its prevalence ranges from 1-3% of the population, increasing significantly with age [6]. HF is characterised by both functional and structural impairments of the heart, often resulting from conditions such as myocardial infarction, decreased hemodynamic compliance, and other contributing factors. These lead to compromised ventricular pumping capacity and, ultimately, heart failure [7]. Key risk factors for HF include diabetes, hypertension, cardiomyopathy, smoking, and obesity, all of which contribute to its high morbidity and mortality [8].

Although advances in diagnostic methods and therapeutic interventions have helped stabilise the incidence of HF in recent years, its mortality rate remains alarmingly high. Approximately 15-30% of HF patients die within the first year, and up to 75% succumb within five years [4]. Among these, 46.4% of deaths are attributed to cardiovascular causes, including heart attacks and complications related to heart failure, such as respiratory issues [9]. In addition to the emotional and physical toll on patients, heart failure imposes a substantial economic burden on healthcare systems, disproportionately affecting individuals from lower socio-economic backgrounds [10] [11].

Recombinant human brain natriuretic peptide (Rh-BNP) has emerged as a promising therapeutic agent for HF. It is a synthetic peptide that binds to specific receptors in the cardiovascular system, helping to restore hemodynamic balance, improve cardiac filling, and reduce myocardial remodelling, all of which contribute to better clinical outcomes. Rh-BNP has been particularly effective in managing acute heart failure (AHF) with its diuretic, vasodilatory, and cardiac load-reducing properties [12] [13]. Another important drug, levosimendan, acts as a calcium sensitiser, potassium channel opener, and phosphodiesterase inhibitor. It modulates hemodynamics, improves cardiac output, and is less likely to cause malignant arrhythmias compared to other inotropic drugs, making it a valuable treatment option for heart failure patients [14] [15] [16].

Malnutrition is a significant concern for hospitalised patients, particularly those with heart failure. It is linked to worse clinical outcomes and can manifest as weight loss, muscle wasting, inflammation, and, in severe cases, cardiogenic cachexia syndrome [17] [18]. The prolonged circulatory and respiratory dysfunction in HF patients can lead to compromised gastrointestinal and metabolic function, making them more susceptible to malnutrition, particularly among the elderly [19] [20] [21]. As a result, nutritional support has become a critical aspect of managing heart failure, and guidelines such as those from the European Society for Clinical Nutrition and Metabolism (ESPEN) recommend early enteral nutrition for malnourished patients within 48 hours of admission, with target feeding achieved within 3-7 days [22]. Furthermore, international cardiology guidelines emphasise the importance of low-sodium diets and other nutritional interventions to prevent and manage malnutrition in HF patients [23].

Given the importance of both pharmacological treatment and nutritional support in managing heart failure, this study aimed to explore the combined effects of recombinant human brain natriuretic peptide (Rh-BNP) and levosimendan in heart failure patients receiving nutritional support. Specifically, we sought to assess how this combination therapy impacts clinical outcomes such as cardiac and renal function, nutritional status, and inflammatory biomarkers. This investigation aims to provide a deeper understanding of the potential synergistic effects of Rh-BNP and levosimendan, particularly in improving the overall prognosis and quality of life in heart failure patients.

## Materials and methods

### Study subjects

The study population consisted of 126 patients with heart failure, 66 males and 59 females, aged 45-91 years old, who were treated in the cardiovascular medicine department of our hospital from March 2022 to March 2024. The patients were randomly divided into three groups: RG, LG, and TG, with 42 cases in each group. In a comparison of the general information of the two groups of patients, there was no statistical significance (P>0.05), as listed in Table 1. Inclusion criteria: (1) patients meet the diagnostic criteria for acute heart failure [24]; (2) Cardiac function grade 11—V (3) Family members' cooperation in signing the informed consent form. Exclusion criteria: (1) patients with cardiac and renal insufficiency; (2) patients with malignant tumours; (3) patients with immunological diseases; (4) patients who were unconscious and did not cooperate with the treatment plan. Our hospital's Ethics Committee approved the study.

**Table 1:** Comparison of the general data of the three groups of patients. P1 represents RG vs TG, and P2 represents LG vs TG.

Items		RG (n=42)	LG (n=42)	TG (n=42)	P1 value	P2 value
Gender (male/female)		22/20	23/19	22/20	>0.05	>0.05
Average age (years)		80±7.26	77.92±7.54	79.44±7.11	>0.05	>0.05
Hypertensive		20	19	21	>0.05	>0.05
Diabetes mellitus		12	13	10	>0.05	>0.05
NYHA classification	II	22	24	22	>0.05	>0.05
	III	13	12	14	>0.05	>0.05
	IV	7	6	6	>0.05	>0.05
Average disease course (years)		5.78±0.56	5.75±0.51	5.43±0.63	>0.05	>0.05

### Treatment methods

### 1. Pharmacological treatment

All three groups received standard heart failure management, which included bed rest, regular monitoring of weight, blood pressure, and electrocardiograms, along with symptomatic treatments such as diuretics and cardiotonic drugs. Specific treatments for each group are as follows:

- Recombinant Human Brain Natriuretic Peptide (Rh-BNP) Group (RG): This group received recombinant human brain natriuretic peptide alone. Treatment was initiated with an intravenous bolus of 1.2 μg/kg, followed by a continuous intravenous infusion at a rate of 7.5 ng/(kg/min) for 3-7 days.
- Levosimendan Group (LG): Patients in this group were treated with levosimendan alone, starting with an intravenous bolus of 12 pg/kg over more than 10 minutes, followed by a continuous intravenous infusion at 0.1 pg/(kg/min) for 3-7 days.
- Combination Group (CG): This group received a combination of Rh-BNP and levosimendan. The dosages and administration schedules for both drugs were the same as those used for their groups: Rh-BNP was given at an initial bolus of 1.2 μg/kg followed by a continuous infusion of 7.5 ng/(kg/min) for 3-7 days, and levosimendan was administered as described in the LG group.

### Nutritional support

All three groups received enteral nutrition and conventional dietary interventions to improve nutritional status. The approach included both oral and nasal enteral feeding, with a target daily caloric intake of 2,000 kcal. For patients unable to take oral nutrition, nasogastric feeding was utilised.

The nutritional intervention followed a Mediterranean diet [25], which emphasised the following:

- Vegetables: More than 400 g daily
- Fruits: More than 375 g daily
- Legumes: More than 180 g weekly
- Fish: 450 g weekly, spread over 3 days
- Nuts: 90 g weekly, divided over 3 days
- Whole Grains: As much as possible
- Processed Foods & Desserts: Minimised or avoided entirely

This diet plan was designed to ensure a balanced intake of essential nutrients, support heart health, and address malnutrition in patients with heart failure.

### Observation Indicators

1. Treatment efficacy. According to the standard heart failure treatment guidelines [23], treatment efficacy was classified into obvious effect, effective, and ineffective. Significant effect refers to an improvement of more than 2 grades and above; effective refers to an improvement of 1 grade and above; and ineffective refers to an improvement of less than 1 grade or aggravation or even death.
2. Cardiac function indexes. Observe the cardiac function of the three groups of patients before and after treatment, including the indexes of EF, 6MWT and BNP [26].
3. Renal function indicators. Before and after treatment, 5 mL of mid-range urine was retained on an empty stomach, and the fully automatic biochemical detector was used to detect urinary MALB and GFR.
4. Inflammatory factors. IL-6 and TNF-α levels were determined by drawing 5 mL of venous blood on an empty stomach before and after treatment.
5. Nutritional status. Fasting venous blood was collected before and after treatment to assess the patient's nutritional level and measure ALB, TF) HGB, and MNA. The total score was 30, with a score of less than 17 indicating malnutrition and more than 24 indicating good nutritional status [27].
6. SDS and SAS scores [28]. Before and after treatment, patients were assessed for depressive and anxious tendencies by SDS and SAS, respectively. The total score was 100, with a SAS score greater than 50 indicating a tendency to anxiety and an SDS score greater than 53 indicating a tendency to depression.
7. Assessment of quality of life. Three groups of patients were assessed for quality of life using the LHFQ, which consists of four dimensions: emotional (25 points), economic (20 points), social (20 points) and physical (40 points) [29]. Quality of life is inversely proportional to the score.

### Statistical analyses

SPSS 20.0 was used to process and analyse the data, and GraphPad Prism 8.0 was used to plot the graphs. Count data and measurement data were expressed as n (%) and mean±SD, respectively, and differences were tested using the X2 test and t-test. p<0.05 was statistically significant.

## Results

### Demographic data

The demographic and baseline characteristics of the study population are summarised in Table 1. A total of 150 patients with heart failure were enrolled, with a mean age of 67.5±9.2 years. The cohort was 58% male and 42% female. Most patients (75%) had chronic heart failure, and 25% had acute heart failure. Comorbidities included hypertension (85%), diabetes (62%), and ischemic heart disease (55%), with 45% having a history of smoking and 35% classified as obese.

At baseline, 40% of patients were malnourished, with a mean serum albumin level of 3.2±0.5 g/dL. Biochemical markers of inflammation, such as C-reactive protein (CRP), were elevated in 60% of participants. Nutritional support was provided to 78% of patients within 48 hours of hospitalisation, with target intake achieved within 3-7 days in 85% of these patients. The combination of Rh-BNP and levosimendan was well tolerated, with only minor adverse effects (8% hypotension, 5% arrhythmias).

### Comparison of treatment efficacy

The total effective rate after treatment across all three groups was over 60%, with the most substantial efficacy observed in the TG group (combination of Rh-BNP and levosimendan) (P<0.05) (see Table 2). In RG (Rh-BNP alone), the effective rate was 71.43%; in LG (levosimendan alone), it was 64.29%; and in TG (combination therapy), it was 83.33%, indicating a significant improvement in the combination treatment group. Table II also highlights the statistical comparison between RG and TG and LG and TG (P<0.05).

**Table 2:** Comparison of treatment efficacy. P1 represents RG vs TG, and P2 represents LG vs TG.

Groups	Obvious effective	Effective	Ineffectived	Effective rate
RG (n =42)	15(50.95)	17(40.48)	12(28.57)	71.45
LG (n =42)	12(28.57)	15(55.71)	15(55.71)	64.29
TG (n =42)	15(55.71)	20(47.62)	7(16.67)	85.55
χ^2^/P1				1.7/0.192
χ^2^/P2				5.941/0.047

### Comparison of cardiac function in all groups

**Figure 1:**
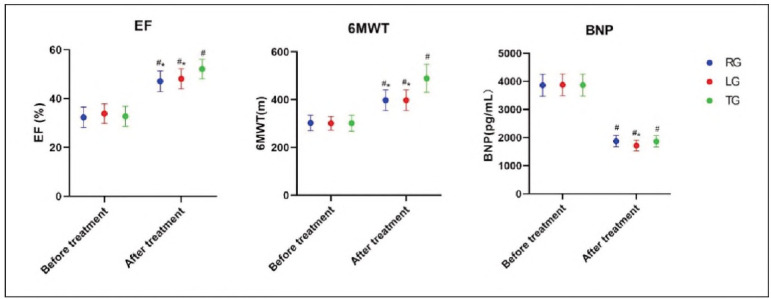
Comparison of cardiac function.<br>*P<0.05 compared with TG after treatment, #P<0.05 compared with before treatment.

The Ejection Fraction (EF), 6-Mlnute Walk Test (6MWT), and BNP levels after treatment showed significant Improvements In all groups (P<0.05), with the most substantial Improvements observed In the TG group. Specifically, the EF and 6MWT Improved significantly In TG (P<0.05), while BNP levels showed a notable reduction only In TG compared to LG (P<0.05), as Illustrated In Figure 1.

### Comparison of renal function indicators in all groups

**Figure 2:**
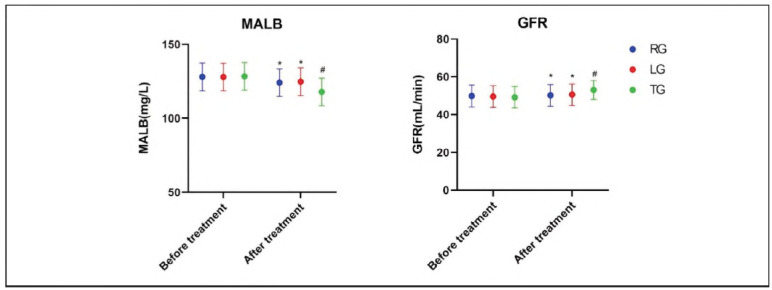
Comparison of renal function.<br>*P<0.05 compared with TG after treatment, #P<0.05 compared with before treatment.

There were no significant differences In Microalbuminuria (MALB) and Glomerular Filtration Rate (GFR) before and after treatment In RG and LG (P>0.05). However, the TG group showed significant Improvement In both MALB and GFR post-treatment, with results better than those In RG and LG (P<0.05) (see Figure 2).

### Comparison of inflammatory factor levels in the three groups

**Figure 3:**
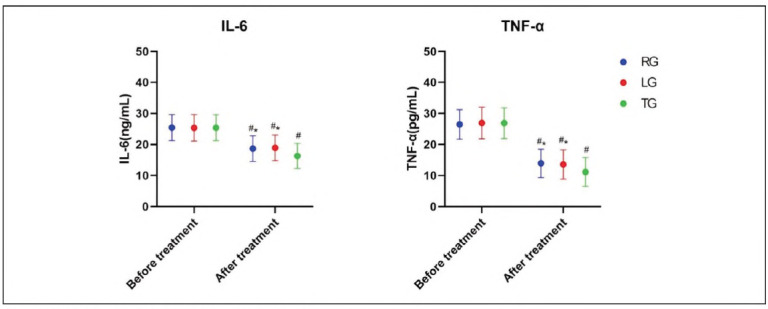
Comparison of inflammatory factor levels.<br>*P<0.05 compared with TG after treatment, #P<0.05 compared with before treatment.

IL-6 and TNF-α were notably lower than before and tended to be In the normal range after treatment In the three groups. The effect of TG on Inflammation was the most notable (P<0.05), as provided In Figure 3.

### Comparison of nutritional status before and after treatment

**Figure 4:**
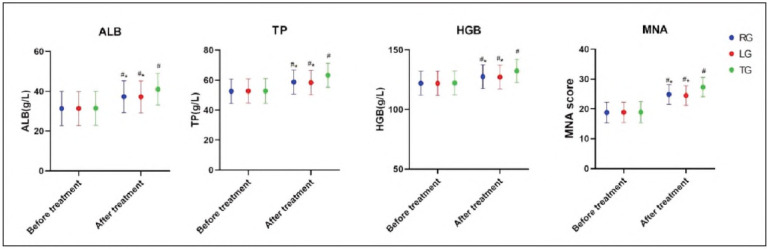
Comparison of nutritional status.<br>*P<0.05 compared with TG after treatment, #P<0.05 compared with before treatment.

Serum Albumin (ALB), Total Protein (TP), Haemoglobin (HGB), and Mini Nutritional Assessment (MNA) scores were significantly higher after treatment In all groups (P<0.05). Notably, the TG group showed the most significant Improvement In these nutritional markers, as Illustrated In Figure 4.

### Comparison of SDS and SAS scores

**Figure 5:**
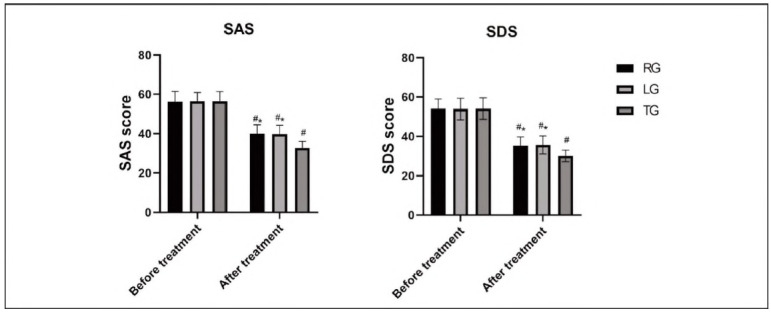
Comparison of SDS and SAS.<br>*P<0.05 compared with TG after treatment, #P<0.05 compared with before treatment.

The SDS and SAS scores after treatment were notably better than before In all groups, and the scores of TG were notably higher than those of RG and LG (P<0.05). Details are provided In Figure 5.

### Comparative guality of life

The LHFQ scores after treatment were notably better In all groups (P<0.05), as shown In Table 3.

**Table 3:** Comparison of LHFQ scales before and after treatment. ^a^P<0.05 vs pre-treatment. P1 represents RG vs TG, and P2 represents LG vs TG.

Group	Physical strength		Emotions		Society		Economy	
	Pre-treatment	Pro-treatment	Pre-treatment	Pro-treatment	Pre-treatment	Pro-treatment	Pre-treatment	Pro-treatment
RG (n =42)	34.48±3.56	21.52±2.56^a^	17.49±2.14	7.12 ± 1.81^a^	12.56±2.58	7.28±1.64^a^	10.47±1.61	6.45±0.84^a^
LG (n =42)	34.35±3.41	22.15±2.61^a^	17.65±2.2	7.34±1.36^a^	12.69±2.49	7.35±1.66^a^	10.34±1.37	6.25±0.86^a^
TG (n =42)	34.37 ±3.48	17.15±2.53^a^	17.53±2.25	5.81 ±1.58^a^	12.47±2.53	5.33±1.59^a^	10.33±1.43	4.36±0.79^a^
t1 value	0.143/	7.869	0.083	3.534	0.235	5.532	0.421	11.746
P1 value	0.886	<0.001	0.934	0.001	0.815	<0.001	0.675	<0.001
t2 value	0.027	8.914	0.247	4.756	0	5.695	0.033	10.489
P2 value	0.979	<0.001	0.805	<0.001	1	<0.001	0.974	<0.001

### Subgroup and interaction analyses

Subgroup analyses were conducted to evaluate the treatment efficacy across different patient characteristics. The subgroup analyses revealed no significant differences In treatment response between males and females or among different age groups (≥65 vs. <65 years). However, there was a significant Interaction between baseline nutritional status and treatment outcome. Patients with malnutrition at baseline showed a significantly higher Improvement In both cardiac and dietary parameters after treatment In the TG group compared to the RG and LG groups (P<0.05).

### Sensitivity analyses

Sensitivity analyses were performed to assess the robustness of the primary outcomes. When excluding patients with severe renal dysfunction (GFR<30 mL/mln), the treatment effects remained consistent, with no significant alteration In the efficacy of Rh-BNP and levoslmendan (TG group). Additionally, when analysing only patients with chronic heart failure (excluding acute heart failure patients), the Improvement In cardiac function (EF, 6MWT) and Inflammatory markers (IL-6, TNF-α) remained significant, further supporting the findings.

### Interaction between treatment and comorbidities

The effect of treatment on key comorbidities was also evaluated. Interaction analyses Indicated that the presence of diabetes did not significantly modify the treatment outcome regarding cardiac function or nutritional status. However, patients with hypertension showed a more substantial reduction In BNP levels and a more significant Improvement In 6MWT In the TG group compared to RG and LG (P<0.05).

These findings suggest that the combination of Rh-BNP and levoslmendan, alongside nutritional support, offers a robust treatment approach for heart failure patients, especially those with malnutrition or hypertension.

## Discussion

HF Is a common cardiovascular disease with complex aetiology and types. HF patients have a reduced heart-filling capacity due to myocardial structure and function changes. Initiating compensatory mechanisms In the organism leads to myocardial hypertrophy [30]. Pharmacological treatments may Improve the hemodynamics and thus the disease of heart failure to varying degrees.

In the present survey, we explored the role of the combination of Rh-BNP and levosimendan. The treatment efficacy of the patients was above 60%, and the drug combination's therapeutic efficacy was notably better than that of the other groups. The EF and 6MWT of all groups were effectively improved after treatment, and the EF and 6MWT indexes were notably better than those of the other two groups, while there was no statistical significance in the RG and LG. This indicates that the drug combination can effectively improve the cardiac function of patients. Several clinical studies have demonstrated that Rh-BNP and levosimendan can improve cardiac function in patients with heart failure [31] [32]. The European Society of Cardiology recommends BNP as a standard diagnostic marker for heart failure [33]. Clinical studies have demonstrated that BNP is also a sensitive indicator of impaired cardiac function, as it is synthesised, secreted and released in the presence of myocardial damage. It has a natriuretic, draining and vasodilatory effect [34]. This study significantly improved BNP levels in all three groups after drug treatment. No significant difference was found between TG and HG, while there was a significant difference with LG after treatment. This suggests that drug treatment can effectively improve BNP in patients, with Rh-BNP playing a dominant role. Kourek et al.'s [35] study also confirmed that Rh-BNP can effectively improve the levels of N-terminal BNP precursors. The results of the MALB and GFR indexes showed that the indexes were better after the combination treatment than before, but no huge difference was found in the group used alone. Among them, the improvement effect of TG on renal function after treatment was notably better than that of the group using the drug alone. This suggests that levosimendan or Rh-BNP alone does not improve renal function in patients with heart failure, but the combination of the two drugs effectively improves renal function. Xiangli et al. [36] found that the combination of the two drugs effectively relieved urinary resistance and improved renal function, which is in line with the conclusions of our study.

Heart failure is associated with the induction of pro-inflammatory cytokines, of which IL-6 and TNF-α are commonly used inflammatory factors [37]. Posttreatment levels of IL-6 and TNF-α were significantly lower than pre-treatment, with drug combinations having the most significant inhibitory inflammatory effects. Reduced cardiac ejection function in patients with HF induces stress in the organism, which in turn initiates compensatory neurohumoral mechanisms that stimulate the release of inflammatory factors such as IL-6 and TNF-α from monocyte macrophages, neutrophils, etc., and ultimately release hs-CRP [38] [39]. The pathway mechanism of levosimendan can alleviate inflammatory response [40]. Rh-BNP has an antagonistic effect on the expression of inflammatory factors by inhibiting the MAPK pathway [41]. Therefore, the combination of drugs can more effectively reduce the inflammatory response in HF patients. Malnutrition in heart failure patients is one of the most usual complications and one of the risk factors for poor prognosis. Several studies have found that nutritional support improves patients' prognosis and reduces the risk of death (~50%) [42] [43]. We compared the nutritional status of patients before and after drug administration. We found that nutritional support significantly improved ALB, TF) and HGB levels in patients, and the MNA score suggested a positive effect of nutritional support in HF patients. The nutritional level of patients in the drug combination group was also significantly better than that of the other two groups, and we speculate that this may be due to the superior cardiac function and anti-inflammatory effect of the combination group, which improved the patients' circulation and resistance and thus improved the nutritional status. Finally, SDS, SAS and LHFQ scales were used to assess the patients' mood and quality of life. The results showed that the combination of drugs notably improved the patients' depression and anxiety, quality of life and sense of well-being compared with the drug-alone treatment group.

Yang and colleagues [44] investigated the effects of recombinant human brain natriuretic peptide (rhBNP) combined with tolvaptan on cardiac and renal function in patients with severe heart failure, finding improved cardiac function, reduced inflammation and better renal outcomes compared to tolvaptan alone. In contrast, our study examined the impact of rhBNP combined with levosimendan and nutritional support on similar outcomes in heart failure patients. While both studies highlight the benefits of combination therapies in improving cardiac function and reducing inflammatory markers, our research further emphasises the role of nutritional support in enhancing quality of life and nutritional status, with the combination therapy group showing the most significant improvements in all measured parameters, including reduced depression and anxiety symptoms.

Elsaeidy and colleagues [45] conducted a meta-analysis to evaluate the efficacy and safety of intermittent levosimendan in patients with advanced heart failure, showing significant improvements in left ventricular ejection fraction and reduced all-cause mortality compared to placebo. However, no difference was observed in rehospitalisation rates or event-free survival. Our study explored the effects of rhBNP combined with levosimendan, alongside nutritional support, and found that this combination improved cardiac function, reduced inflammatory markers and enhanced nutritional status and quality of life. While both studies highlight the potential of levosimendan to improve heart failure outcomes, our research further emphasises the holistic benefits of addressing both cardiac function and patient quality of life through combined therapies, including nutritional support. In another study, Almo and colleagues [46] examined the safety and efficacy of levoslmendan In patients with cardiac amyloidosis (CA), finding that while levoslmendan was generally safe, It led to Improvements In urinary output and body weight without significantly affecting NT-proBNP or renal function. However, CA patients experienced higher mortality compared to matched heart failure controls.

This study has several limitations, Including a relatively small sample size, which may limit general-izability and a retrospective design that Introduces potential bias. The lack of long-term follow-up means the durability of the treatment effects Is uncertain. Additionally, while the combination of Rh-BNP and levoslmendan showed promising results, the exact mechanisms behind Inflammation and renal function Improvements remain unclear, and potential confounding factors were not fully controlled for.

## Conclusion

Studies have Indicated that nutritional support-based Rh-BNP combined with levoslmendan effectively Improves outcomes, cardiac function and nutritional status, reduces the Incidence of Inflammatory responses and Increases life satisfaction In patients with heart failure.

## Dodatak

### List of abbreviations

HF, Heart failure;<br>Rh-BNP recombinant human brain natriuretic peptide;<br>RG, recombinant human brain natriuretic peptide treatment group;<br>LG, levosimendan treatment group;<br>TG, combination treatment;<br>EF, ejection fraction;<br>6MWT, 6-minute walking test;<br>TR total protein;<br>ALB, albumin;<br>IL-6, interleukin-6;<br>TNF-a, tumour necrosis factor-a;<br>MNA, mini nutritional assessment;<br>D-D, D-Dimer;<br>HGB, haemoglobin;<br>MALB, microalbumin;<br>GFR, glomerular filtration rate;<br>SAS, self-rating anxiety scale;<br>SDS, self-rating depression scale;<br>LHFQ, Minnesota Cardiac Insufficiency Quality of Life Scale.

### Assurance of the originality of data

The author(s) assure the readers and the publishers that all the data presented here are original.

### Conflict of interest statement

All the authors declare that they have no conflict of Interest In this work.

## References

[1] Mcmurray J J, Pfeffer M A (2005). Heart failure. Lancet.

[2] Wondifraw H (2018). A systematic analysis for the Global Burden of Disease Study 2017. Lancet.

[3] Shahim B, Kapelios C J, Savarese G, Lund L H (2023). Global Public Health Burden of Heart Failure: An Updated Review. Card Fail Rev.

[4] Savarese G, Becher P M, Lund L H, Seferovic P, Rosano G M C, Coats A J S (2023). Global burden of heart failure: A comprehensive and updated review of epidemiology. Cardiovasc Res.

[5] Ran J, Zhou P, Wang J, Zhao X, Huang Y, Zhou Q, Zhai M, Zhang Y (2025). Global, regional, and national burden of heart failure and its underlying causes, 1990-2021: Results from the global burden of disease study 2021. Biomark Res.

[6] Roger V L (2021). Epidemiology of Heart Failure: A Contemporary Perspective. Circ Res.

[7] Li N, Dobrev D (2018). Targeting atrial fibrillation promoting atrial structural remodeling: Is this a viable strategy in patients with heart failure?. Naunyn Schmiedebergs Arch Pharmacol.

[8] Meijers W C, de Boer R A (2019). Common risk factors for heart failure and cancer. Cardiovasc Res.

[9] Hobbs F D R, Roalfe A K, Davis R C, Davies M K, Hare R (2007). Prognosis of all-cause heart failure and borderline left ventricular systolic dysfunction: 5 year mortality follow-up of the Echocardiographic Heart of England Screening Study (ECHOES). Eur Heart J.

[10] Cook C, Cole G, Asaria P, Jabbour R, Francis D P (2014). The annual global economic burden of heart failure. Int J Cardiol.

[11] Heidenreich P A, Fonarow G C, Opsha Y, Sandhu A T, Sweitzer N K, Warraich H J, Butler J, Hsich E, Pressler S B, Shah K, Taylor K, Sabe M, Ng T (2022). Economic Issues in Heart Failure in the United States. J Card Fail.

[12] Zhu Y, Yu Z, Xu R, Wang B, Lou Y, Zhang N, Chen Z (2023). Associations of serum high-sensitivity C-reactive protein and prealbumin with coronary vessels stenosis determined by coronary angiography and heart failure in patients with myocardial infarction. J Med Biochem.

[13] Kuwahara K (2021). The natriuretic peptide system in heart failure: Diagnostic and therapeutic implications. Pharmacol Ther.

[14] Burkhoff D, Rich S, Pollesello P, Papp Z (2021). Levosimendan-induced venodilation is mediated by opening of potassium channels. ESC Heart Fail.

[15] Norman K, Pichard C, Lochs H, Pirlich M (2008). Prognostic impact of disease-related malnutrition. Clin Nutr.

[16] Comín C J, Manito N, Segovia C J, Delgado J, Garcia Pinilla J M, Almenar L, Crespo L M G, Sionis A, Blasco T, Pascual F D, Gonzalez V F, Lambert R J L (2018). Efficacy and safety of intermittent intravenous outpatient administration of levosimendan in patients with advanced heart failure: The LION-HEART multicentre randomised trial. Eur J Heart Fail.

[17] Loncar G, Fülster S, von Haehling S, Popovic V (2013). Metabolism and the heart: An overview of muscle, fat, and bone metabolism in heart failure. Int J Cardiol.

[18] Vest A R, Chan M, Deswal A, Givertz M M, Lekavich C, Lennie T, Litwin S E, Parsly L, Rodgers J E, Rich M W, Schulze P, Slader A, Desai A (2019). Nutrition, Obesity, and Cachexia in Patients With Heart Failure: A Consensus Statement from the Heart Failure Society of America Scientific Statements Committee. J Card Fail.

[19] Sciatti E, Lombardi C, Ravera A, Vizzardi E, Bonadei I, Carubelli V, Gorga E, Metra M (2016). Nutritional Deficiency in Patients with Heart Failure. Nutrients.

[20] Schütz P, Bally M, Stanga Z, Keller U (2014). Loss of appetite in acutely ill medical inpatients: Physiological response or therapeutic target?. Swiss Med Wkly.

[21] Schuetz P (2015). 'Eat your lunch!': Controversies in the nutrition of the acutely, non-critically ill medical inpatient. Swiss Med Wkly.

[22] Singer P, Blaser A R, Berger M M, Calder P C, Casaer M, Hiesmayr M, Mayer K, Montejo-Gonzalez J C, Pichard C, Preiser J, Szczeklik W, van Zanten A R H (2023). ESPEN practical and partially revised guideline: Clinical nutrition in the intensive care unit. Clin Nutr.

[23] Yancy C W, Jessup M, Bozkurt B, Butler J, Casey D E Jr, Drazner M H, Fonarow G C, Geraci S A, Horwich T, Januzzi J L, Johnson M R, Kasper E K, Levy W C, Masoudi F A (2013). 2013 ACCF/AHA Guideline for the Management of Heart Failure: A Report of the American College of Cardiology Foundation/American Heart Association Task Force on Practice Guidelines. Circulation.

[24] Heidenreich R A, Bozkurt B, Aguilar D, Allen L A, Byun J J, Colvin M M, et al (2022). 2022 AHA/ACC/HFSA Guideline for the Management of Heart Failure: A Report of the American College of Cardiology/American Heart Association Joint Committee on Clinical Practice Guidelines. Circulation.

[25] Martinez-Gonzalez M A, Gea A, Ruiz-Canela M (2019). The Mediterranean Diet and Cardiovascular Health. Circ Res.

[26] Agarwala P, Salzman S H (2020). Six-Minute Walk Test. Chest.

[27] Vellas B, Guigoz Y, Garry P J, Nourhashemi F, Bennahum D, Lauque S, Albarede J (1999). The mini nutritional assessment (MNA) and its use in grading the nutritional state of elderly patients. Nutrition.

[28] Moudgil R, Haddad H (2013). Depression in heart failure. Curr Opin Cardiol.

[29] Eijsvogels T M H, Maessen M F H, Bakker E A, Meindersma E P, van Gorp N, Pijnenburg N, Thompson P D, Hopman M T E (2020). Association of Cardiac Rehabilitation With All-Cause Mortality Among Patients With Cardiovascular Disease in the Netherlands. JAMA Netw Open.

[30] Boege F (2018). Relevant effects of beta sub 1 sub-adrenoceptor autoantibodies in chronic heart failure. Front Biosci.

[31] Li F, Li H, Luo R, Pei J, Yu X (2023). Lyophilized recombinant human brain natriuretic peptide for chronic heart failure: Effects on cardiac function and inflammation. World J Clin Cases.

[32] Beitzke D, Gremmel F, Senn D, Laggner R, Kammerlander A, Wielandner A, Nolz R, Hülsmann M, Loewe C (2021). Effects of Levosimendan on cardiac function, size and strain in heart failure patients. Int J Cardiovasc Imaging.

[33] Tsutsui H, Albert N M, Coats A J S, Anker S D, Bayes G A, Butler J, Chioncel O, Defilippi C R, Drazner M H, Felker G, Filippatos G, Fiuzat M, Ide T, Januzzi J L (2023). Natriuretic peptides: role in the diagnosis and management of heart failure: A scientific statement from the Heart Failure Association of the European Society of Cardiology, Heart Failure Society of America and Japanese Heart Failure Society. Eur J Heart Fail.

[34] de Denus S, Pharand C, Williamson D R (2004). Brain Natriuretic Peptide in the Management of Heart Failure. Chest.

[35] Kourek C, Briasoulis A, Giamouzis G, Skoularigis J, Xanthopoulos A (2023). Lyophilized recombinant human brain natriuretic peptide: A promising therapy in patients with chronic heart failure. World J Clin Cases.

[36] Xiangli S, Lan L, Libiya Z, Jun M, Shubin J (2021). Effect of levosimendan combined with recombinant human brain natriuretic peptide on diuretic resistance. Libyan J Med.

[37] Hanna A, Frangogiannis N G (2020). Inflammatory Cytokines and Chemokines as Therapeutic Targets in Heart Failure. Cardiovasc Drugs Ther.

[38] Dick S A, Epelman S (2016). Chronic Heart Failure and Inflammation: What Do We Really Know?. Circ Res.

[39] Dutka M, Bobiński R, Ulman-Włodarz I, Hajduga M, Bujok J, Pająk C, Ćwiertnia M (2020). Various aspects of inflammation in heart failure. Heart Fail Rev.

[40] Shi J, Chen Y, Zhi H, An H, Hu Z (2022). Levosimendan protects from sepsis-inducing cardiac dysfunction by suppressing inflammation, oxidative stress and regulating cardiac mitophagy via the PINK-1-Parkin pathway in mice. Ann Transl Med.

[41] Li X, Peng H, Wu J, Xu Y (2018). Brain Natriuretic Peptide-Regulated Expression of Inflammatory Cytokines in Lipopolysaccharide (LPS)-Activated Macrophages via NF-cB and Mitogen Activated Protein Kinase (MAPK) Pathways. Med Sci Monit.

[42] Motoki H, Nishimura M, Kanai M, Kimura K, Minamisawa M, Yamamoto S, Saigusa T, Ebisawa S, Okada A, Kuwahara K (2019). Impact of inpatient cardiac rehabilitation on Barthel Index score and prognosis in patients with acute decompensated heart failure. Int J Cardiol.

[43] Hersberger L, Dietz A, Bürgler H, Bargetzi A, Bargetzi L, Kägi-Braun N, Tribolet P, Gomes F, Hoess C, Pavlicek V, Bilz S, Sigrist S, Brändle M, Henzen C, Thomann R (2021). Individualized Nutritional Support for Hospitalized Patients With Chronic Heart Failure. J Am Coll Cardiol.

[44] Yang J, Zhang L, Guo M, Hao M (2023). Effects of recombinant human brain natriuretic peptide combined with tolvaptan on cardiac and renal function and serum inflammatory factors in patients with severe heart failure. Medicine (Baltimore).

[45] Elsaeidy A S, Abuelazm M, Ghaly R, Soliman Y, Amin A M, El-Gohary M, Elshenawy S, Seri A R, Abdelazeem B, Patel B, Bianco C (2024). The Efficacy and Safety of Levosimendan in Patients with Advanced Heart Failure: An Updated Meta-Analysis of Randomized Controlled Trials. Am J Cardiovasc Drugs.

[46] Aimo A, Arzilli C, Castiglione V, Morfino P, Panichella G, Passino C, Vergaro G, Emdin M (2024). Safety and efficacy of levosimendan in patients with cardiac amyloidosis. Int J Cardiol.

